# The Contact Conundrum: Are We Introducing Contact at the Correct Time in Youth Sports?

**DOI:** 10.1007/s40279-025-02348-6

**Published:** 2025-12-04

**Authors:** Stephen W. West, Sharief Hendricks, Sean P. Cumming, Kathryn Dane, Siobhán O’Connor, Ben Jones, Brooke Patterson, Ash T. Kolstad, Steven Broglio, Carolyn A. Emery, Carly D. McKay

**Affiliations:** 1 Centre for Health, and Injury and Illness Prevention in Sport, Department for Health, University of Bath, Bath, UK; 2 UK Collaborating Centre on Injury and Illness Prevention in Sport, Bath, UK; 3 Sport Injury Prevention Research Centre, Faculty of Kinesiology, University of Calgary, Calgary, Canada; 4 Division of Physiological Sciences and Health through Physical Activity, Lifestyle and Sport, Department of Human Biology, Faculty of Health Sciences, University of Cape Town, Cape Town, South Africa; 5 Carnegie Applied Rugby Research (CARR) Centre, Carnegie School of Sport, Leeds Beckett University, Leeds, UK; 6 School of Health and Human Performance, Dublin City University, Dublin, Ireland; 7 School of Behavioural and Health Sciences, Faculty of Health Sciences, Australian Catholic University, Brisbane, QLD Australia; 8 Premiership Rugby, London, UK; 9 England Performance Unit, Rugby Football League, Manchester, UK; 10 La Trobe Sport and Exercise Medicine Research Centre, La Trobe University, Melbourne, VIC Australia; 11 University of Michigan Concussion Center, University of Michigan, Ann Arbor, MI USA; 12 Alberta Children’s Hospital Research Institute, University of Calgary, Calgary, Canada; 13 Hotchkiss Brain Institute, University of Calgary, Calgary, Canada; 14 Department of Community Health Sciences, Cumming School of Medicine, University of Calgary, Calgary, Canada; 15 Department of Pediatrics, Cumming School of Medicine, University of Calgary, Calgary, Canada; 16 O’Brien Institute for Public Health, University of Calgary, Calgary, Canada; 17 Podium Analytics, London, UK; 18 Stellenbosch Institute for Advanced Study, Wallenberg Research Centre, Stellenbosch, South Africa

## Abstract

Participation in sport offers numerous physiological, psychological, and social benefits, yet injury remains an inherent risk, particularly in collision-based sports. Increasing scrutiny surrounds these sports, especially for youth, with inconsistency in the age for introducing deliberate contact (e.g., body checking, tackle) and debate regarding proposals for banning high-risk actions to reduce injuries. This article explores the policies and controversies regarding how, and when, physical contact is introduced in sports. Current policies vary significantly across sports, sexes, and national jurisdictions, leading to inconsistent implementation and outcomes. We outline arguments for both delaying and lowering the contact introduction age, including implications for participation rates, skill acquisition, and injury risk. Raising the age may reduce injury history and cumulative head impacts, while earlier, progressive contact training may enhance technical competence. Growth, maturation and size discrepancies further complicate such policy decisions. Evidence supports multimodal approaches, including training guidelines (e.g., reduced contact in practices), neuromuscular training, and rule modifications, to enhance safety without compromising play. Weight-based categorisation and bio-banding (grouping players by attributes associated with growth and/or maturation instead of chronological age) strategies show potential for injury-risk reduction but lack comprehensive evaluation. Despite polarised opinions, developing sport-specific recommendations on best practices for contact introduction remains critical to ensuring athlete welfare and sustainable participation in collision sports.

**Supplementary Information:**

The online version contains supplementary material available at 10.1007/s40279-025-02348-6.

## Key Points

**Table:** 

Inconsistent policies: contact introduction in youth sports varies widely across sports, sexes and countries, leading to inconsistent safety and development outcomes.
Early versus late contact debate: delaying contact may reduce injury risk, while early exposure with proper training could improve safety and skill development. Evidence remains inconclusive.
Need for a structured approach: a progressive, evidence-based method, including a multifaceted approach to player welfare, is crucial for safe and effective contact introduction.

## How Did We Get Here?

Participation in sport is widely acknowledged as beneficial for physiological, psychological and social health and wellbeing. Yet, injury related to participation is an unfortunate and sometimes unavoidable consequence with more than one in four youth seeking medical attention for a sports injury annually [1]. Injuries can lead to short- and long-term consequences for sport participants, their teams and their families. Therefore, the goal of injury prevention research is to monitor and improve athlete health while encouraging safe and competitive play for all participants. In recent years, collision-based sports (particularly for children and adolescents) have been under increasing scrutiny. This includes calls for bans on specific actions such as body checking in ice hockey [2], tackling in rugby [3] and Australian Rules football [4], as well as banning certain collision sports in specific contexts [5]. More recently, some have gone as far as to suggest that brain trauma induced by participation in ‘impact sports’ could be classified as child abuse [6]. While these views are not shared by all and often provoke a strong response from those engaged in the discussion, concerns over the future of collision sports are apparent, with participation rates in some collision sports stagnating or dropping [7, 8]. This indicates a shift in participation patterns from ‘higher-risk’ sports to ‘lower-risk’ alternatives (e.g., 13.2, 17.5, 26.2 and 23.2% decline in tackle football, ice hockey, lacrosse and wrestling, respectively, with 32.6, 4.3 and 51.2% increase in golf, soccer and tennis, respectively) [9]. Outside of formal sport, there has also been a reduction in the amount of ‘risky outdoor play’ to which children are exposed [10]. In light of these concerns, collision sport governing bodies have broadly been open to evidence-informed change, for example introducing age-based guidelines in many sports or allowing and disallowing certain actions (e.g., disallowing body checking in games in child and some levels of adolescent ice hockey in Canada and the USA, and disallowing contested scrums in rugby before the under-15 (U15) age group in Canada [11]). Beyond the discourse surrounding risk of injury, each year there are tragic cases of catastrophic injuries and fatalities in sport [12, 13]. These events are rare (all traumatic injuries: 0.28/100,000 participants; fatalities: 0.05/100,000 participants [14]), but further raise concerns related to sport participation. In response, several policies, rule changes or stricter adjudication or sanctions (e.g., for dangerous tackles [15, 16]) have been implemented to ensure athlete safety while maintaining the fundamental principles of the sport (e.g., Rowan’s Law [12], “If in doubt, sit them out” [13]).

The rise of media coverage and scrutiny of sport injuries has led to an increase in awareness and recognition of specific injury types, often characterised by their high rates (e.g., concussion) or high severity (e.g., anterior cruciate ligament (ACL) injuries). This is particularly apparent in women’s sport, which has experienced significant growth and media attention, and consequently surging interest in injuries which appear to be common (e.g., ACL injuries). More broadly, concussion is now one of the most extensively researched areas in sport medicine, with substantial growth of knowledge related to prevention, diagnosis, management and treatment [17]. However, guidelines, policies and rule changes in place to protect athletes appear to lack consistency between and within sports. For example, the age at which intentional physical ‘contact’ should be introduced divides opinion within scientific and sporting communities. Some inconsistencies may relate to how ‘contact’ is perceived and represented—for instance, using the term ‘contact’ may suggest incidental collision and a lack of intent or control, with little or no responsibility for the player. From a skill perspective, collision sports require opposing players to intentionally physically engage (contact) each other during each offensive-defensive cycle. The ability to do this is a pre-requisite for safe participation and success, and it is well understood that, for example, the tackle in rugby or body check in ice hockey requires technical skill, and specific physical and psychological qualities. In other words, the ability to make contact is a movement skill, which can be acquired, developed and mastered to protect the player and enhance participation in the sport. Cognitive development also has variable timescales, and can influence young athletes’ abilities to assess risk, learn and execute complex dynamic tasks, and evaluate long-term consequences [18, 19]. This has implications for when and how contact should be introduced. Supporting such skill development is part of the duty of care that players, parents, coaches and sporting bodies have to provide safe participation.

Despite the reported positive effects of participation in risky play [10], there continues to be growing concern around injury risk, repeated head impact exposure, diagnosed concussions, and the long-term consequences of participation in collision sports. As such, in this article we outline (i) current policies for introducing contact and what influences these policies, (ii) considerations for policy makers designing introduction to contact guidelines, and (iii) benefits of an earlier versus later introduction to contact. To do this, evidence pertaining to both the benefits and weaknesses of the current literature have been examined, with the aim of achieving a balanced approach that outlines the challenges associated with decision making in this space.

## What are the Current Policies?

For this article, several sports were assessed for the age at which contact is introduced in either a modified or non-modified format. There was no agreement between, or in many cases within, sports as to the age to introduce contact or in what format (Table 1 and Electronic Supplementary Material (ESM) Table S1). Across sports, age of introduction ranged from U6 to U15, while the sports with the greatest range of ages at which the contact could be introduced were rugby union and Tackle Football (American and Canadian Football). Beyond the heterogeneity in sports, both national and international bodies adopted different sets of rules and, in many cases, differences also existed between sexes (Table 1 and ESM Table S1). Furthermore, the degree to which contact was restricted was also variable, with some modifications including disallowing body checking in games in certain adolescent ice hockey contexts, making deliberate contact in some Gaelic Games, tackling to ground in Australian Rules football, and, more recently, restrictions on making contact above certain heights in some rugby union and rugby league contexts.

**Table 1:** Summary of current policies for competition across ages, sexes and levels of contact across a selection of collision sports

Sport	Consistent age of introduction	Range of age (years) groups	Consistent rules between sexes	Mixed age grades permitted	Type of introduction of contact (i.e., restricted/full-contact)
Rugby union	No	U8-U12	Yes	Country specific	Restricted
Rugby league	No	U6-U8	Yes	Country specific	Restricted
Australian Rules football^a^	No	U10-U11	No	Until U14	Restricted
Gaelic Football (men’s)^a^	Yes	No age restriction	No	Until U12	Full-contact
Hurling^a,b^	Yes	No age restriction	No	Until U12	Full-contact
Box lacrosse	Yes	U6-U15	No	Until U17	Full-contact
Field lacrosse	Yes	U15	No	Until U17	Full-contact
Ice hockey	Yes	U15	No	Until U18	Full-contact
Tackle football	No	U7-U11		Until U18	Full-contact

See Electronic Supplementary Material Table S1 for full details

*U* under

^a^Subject to local guidelines at under 12 years and lower age grades

^b^Male version with Camogie the female alternative

## Considerations for Policy Makers When Designing Introduction to Contact Guidelines

Several complex factors should be considered when developing policies related to the age at which young athletes should be introduced to contact, including the demands of the sport, risk for injury and/or physical harm, rule modifications/adjudication/enforcement, and multimodal complementary approaches and developmental readiness [20].

### Growth and Maturation

Two biological processes commonly cited as risk factors for injury in collision sports are growth and maturation [21]. Whereas growth refers to changes in body size (height, mass), maturation refers to the process of progress towards the adult and/or mature state [22]. Maturation can be defined in terms of status, tempo or timing, and has a well-documented association with athletic performance and selection in sport [23, 24]. To illustrate, for male athletes, advanced maturity status during adolescence is typically associated with significant advantages in size, strength, speed, power and momentum [25]; however, contrary evidence suggests that physical size alone may not relate to superior speed and power performance in Australian rugby union boys [26]. Elsewhere, differences of up to 6 years in skeletal age, an established proxy of biological maturation in youth, have been documented among adolescent athletes of the same chronological age [27, 28]. Ironically, it is at the transition between late childhood and early adolescence that many sports introduce contact, coinciding with the developmental stage where maturity-associated differences in size and athleticism start to emerge [29–32]. As the age at which children enter puberty varies considerably, this increases the likelihood of creating mismatches in size and athleticism between same-age peers, often described as ‘David versus Goliath’ scenarios. While little research has explored differences in relative size for injured players, self-reported injuries by athletes playing ice hockey, tackle football, lacrosse and rugby union suggest that the majority of injuries are caused by players perceived to be larger than themselves, with very few occurring as a result of contact with a smaller player [33].

To combat the potential for size discrepancies in sports, the practice of grouping young athletes by a combination of age- and weight-based criteria is routine in most combat sports, including boxing, wrestling, judo, taekwondo, karate and mixed martial arts [29]. Age- and weight-based competition divisions are designed to promote competitive equity, optimal challenges, and to protect the health and safety of young athletes. Although such categories can account for developmental differences in size, there is evidence to suggest that maturity-associated differences in strength and power can be observed among athletes competing within the same age and weight divisions [34, 35].

### Bio-banding

Grouping athletes based on age- and weight/maturity-based criteria is less common in collision sports, although there are some examples. In American Football, ‘Pop Warner’ leagues have adopted weight banding, with players eligible to play within certain weight brackets for their age (e.g., 8- to 10-year-olds weighing 60–120 lbs and 11-year-olds weighing 60–100 lbs may play in Junior Pee Wee leagues, while 9- to 11-year-olds weighing 75–135 lbs and 12-year-olds weighing 75–110 lbs may play in Pee Wee leagues [36]). Furthermore, some Pee Wee football leagues have introduced a weight-based ball carrier’s rule to determine which players are eligible to carry the ball beyond the line of scrimmage [29]. Several rugby contexts have also adopted weight-grading strategies (e.g., U9 and U10 players being grouped into games for those under 35 kg, under 40 kg, under 50 kg and under 60 kg [37]) or weight-based dispensation policies to allow players to play outside of their regular chronological age bracket in special circumstances [38]. However, these examples are the exception rather than the norm and demonstrate inconsistency in policy design and implementation across leagues, organisations and countries. In some cases, regional and national policies vary. Perhaps the most well-known example is the restricted weight-grade age groups implemented by Auckland Rugby (union) and Auckland Rugby League in New Zealand, which operate both open and weight-restricted age groups to cater for smaller and/or later developing youth. These rules were introduced to accommodate individual and ethnic differences in size and maturation [39, 40].

Research pertaining to the effectiveness of age- and weight-based strategies for enhancing the participation, enjoyment, development, safety or retention of youth athletes is limited; however, emerging evidence suggests potential benefits and costs. Although a recent study of weight-based criteria for assigning junior rugby players to competition bands suggested higher dropout for larger/earlier maturing players required to play up an age group, there appears to be an opposite effect for smaller/later maturing players participating in weight-restricted divisions [41]. As a greater number of participants compete in the weight-restricted divisions than are required to play up an age group, this likely yields a net benefit, but also highlights a need to better support players who may be larger or more advanced in maturity for their age. Furthermore, the use of bio-banding approaches may not only support later maturing athletes, but also addresses higher dropout rates in females compared to their male counterparts [42]. Similarly, a qualitative study of New Zealand youth participating in weight-restricted rugby union leagues indicated that many would not be participating in rugby, or sport at all, if they had been required to compete in open age groups [39].

The benefits of periodically matching athletes based on maturation rather than age, referred to as bio-banding, have been investigated in several sports including soccer [43–46], basketball [47], handball [48] and, more recently, ice hockey [49]. Bio-banding in these sports is used as an adjunct rather than a replacement for age-group competition. This appears to benefit both early and late maturing youth, albeit differentially. Competing against older yet physically matched youth, early maturing players are exposed to greater challenges, required to make decisions more quickly, scan more frequently, play more as a team, and place a greater emphasis upon their tactical and technical attributes [45]. Late maturing players describe the experience of competing against younger yet physically matched peers as less challenging, but they note greater opportunity to demonstrate their technical, tactical and physical attributes, and take on positions of leadership [45]. Notably, both early and later maturing soccer players described bio-banded games as less physically oriented and more technical and tactically oriented, with greater emphasis upon short passing and teamwork, an observation that has been corroborated in performance data [44]. From an injury perspective, both late and early maturing players described the bio-banded games as less dangerous, though there are no objective data to support this perception [45]. More limited studies of bio-banding in basketball [47] and ice hockey [49] suggest similar benefits for both early and late maturing youth in these sports. Despite the adoption of some of these weight-based or bio-banded programs, few have been evaluated for their effectiveness, with one study in American Football suggesting no difference in all injury, non-time-loss injury, time-loss injury or concussion outcomes between age-only and age-and-weight conditions [50].

There are several challenges associated with introducing weight-based or bio-banded divisions into collision sports. Firstly, a recent study highlighted the challenges in estimating maturation status for Gaelic Football coaches, suggesting that an inability to make these assessments may make the feasibility of such programs difficult [51]. Secondly, having enough athletes, coaches, administrators and resources to effectively deliver and manage such programs effectively is a barrier. Less densely populated communities may not have enough participants to implement stratified leagues, and these programs require access to individuals qualified to assess and interpret size and/or maturity data. A potential solution with respect to population size is limiting the number of players competing on each team; however, this would significantly modify the nature and demands of the game and/or sport of interest. Additionally, from the onset of puberty, smaller and/or late maturing players are increasingly less likely to be represented in athletic populations [20], limiting numbers of players for specific weight or maturity bands. Indeed, a recent study in Gaelic Football observed only one player in a sample of ~ 250 was classified as ‘late maturing’ [52]. This suggests that late maturing players, who are potentially smaller and lighter, are not selected for talent development programs. Accordingly, any strategies to address conflicts in size or maturity would need to be implemented before weight and/or maturity discrepancies emerge (i.e. prior to puberty) [20].

### Other Rule Modifications, Adjudication and Enforcement

A strategy that is often suggested as a potential mechanism for reducing risk of injury in collision sports is the removal of contact altogether [3]. While this approach is considered extreme by many and often met with resistance from the sporting community, the topic is revisited regularly. What is often not considered in this debate is whether contact removal or restriction leads to the desired outcomes. For example, in girl’s and women’s ice hockey and ringette, contact is prohibited at all levels of play. Yet, video analysis of in-game physical contact shows that body checking and head contacts occur at rates that are similar to boys’ ice hockey, which allows body checking [53, 54]. As such, the removal of contact from these sports may not actually lead to reductions in contact and subsequent injury rates. It must also be acknowledged that low levels of rule enforcement likely contribute to the lack of effectiveness of prevention strategies in some contexts [53, 55].

### A Multimodal Approach

Beyond altering the age of contact introduction, sports have used a multi-modal approach to minimise risk in contact events across the spectrum of primary prevention (i.e. policies/law changes, protective equipment and training programs [56]). Many of the strategies suggested to reduce the risk of injury [56] and/or concussion do not target contact-related injuries specifically, though targeting collision- and tackle-related injuries has been highlighted as a priority [57]. The strategies that have previously shown the greatest effectiveness for reducing injury rates include disallowing body checking in ice hockey games, limiting contact in American Football, and neuromuscular training programs in rugby [56].

A commonly suggested solution is the concept of multimodal exercise-based training programs to improve physical ability to make contact and reduce the risk of potential injury. These have been introduced in several collision sports where bespoke resources and tools have been developed. For example in Australian Rules football, the national governing body co-created Prep-to-Play [58] with injury prevention and coaching experts, which includes a contact skill training program (e.g., tackling, ground and aerial contests), a sport-specific neuromuscular warm-up program (e.g., includes falling, bumping), and a strength program (e.g., includes core strength) [59]. In rugby union, the global governing body (World Rugby) has introduced two programs called ‘Contact Confident’ [60] and ‘Tackle Ready’ [61], while national governing bodies have their own tools specific to their contexts (e.g., New Zealand [62], South Africa [63] and Canada [64]). In rugby league, the ‘TackleReady’ program has also been introduced [65], while in American Football, programs are offered across a host of contact skills including helmetless and tackle training [66]. While these programs provide coaches with an array of tools and resources, there are several challenges associated with their use, including low adoption, resource availability, and, most importantly, a limited evidence base to support their efficacy and/or effectiveness. Another method that has been proposed to minimise the risk of injury, which has yet to be evaluated, are policies outlining a minimum number of sessions before participation in competitive matches (e.g., all players must participate in ten sessions in Youth Rugby in some contexts [67]); however, the quality of these sessions, as well as enforcement of the policy, have yet to be investigated. While it has been shown that lower tackle proficiency is associated with worse injury outcomes [68], future research should specifically investigate the effectiveness of implementing such tackle programs on future injury risk [69].

Alongside age-related guidelines or policies, law changes have been particularly effective methods in reducing injury and concussion risk, which may be implemented alongside a complementary change in the contact introduction age. For example, in ice hockey, research informed a policy change that disallowed body checking in games in U13 leagues nationally (USA 2011, Canada 2013) and in non-elite levels of play (participants in the lower 60% of divisions) in older age groups (ages 13–17 years) provincially and/or regionally in Canada since 2014 [2]. Since then, similar policy changes have shown significant reductions in injury/concussion rates across several age groups [70–72]. The appropriate age and level of play for body checking to be introduced in games has been a topic of discussion for over 30 years. Beyond injury rates alone, consideration of the adoption of new player behaviour patterns is also important, with Martinez et al. [73] reporting lower intensity impacts, player contacts and head impacts using video analysis. Importantly, when considering implementation of rule and policy changes to impact player welfare outcomes, to ensure stakeholder buy-in, performance-related outcomes must also be considered. Body-checking policies have also shown no negative consequences on offensive performance (e.g., shots on goal/completed offensive passes [74]); however, more research is needed to consider other player behaviours, game strategies, and player performance.

In American Football, a range of measures including reduced equipment use (71% reduction), reduced contact practices (10% reduction—not significant), reduced intensity practices (81% reduction) and restricted collision time (70% reduction) have been shown to result in a net 64% reduction in injury risk [56]. Despite these positive findings, the most critical limitation within the current evidence base is the absence of literature related to female/women and girl athletes [56, 75].

### Implementation Strategies

Sport governing bodies should consider strategies to ensure interventions (e.g., policies/law changes, protective equipment and/or training programs) are implemented and sustained over time. For example, coaches may lack confidence to implement tackle-training, and desire more education and support to design appropriate tackle drills, constraints and periodisation for their context. Strategies may include dedicated sport-system resources to provide technical support or auditing, or annual education and/or workshops integrated into existing operations (e.g., coach accreditation/courses [59, 76]).

## Benefits of Introducing Contact at a Later Age

### Participation Rates

A later introduction to contact could have several benefits for participation rates. Firstly, players (and their parents) who have apprehension about participating may feel more at ease with a later introduction of contact. Secondly, injury has been reported to account for half of all drop-outs from youth and junior athletics [77], while an estimated 8% of Australian adolescents drop out of playing sport due to injury, or fear thereof [78]. A delayed and/or progressive introduction to contact may lead to a reduction in injury rates, promoting greater retention of players in the game to senior levels.

### Reduction in Overall Injury

Reducing injury rates in adolescent players may have a significant effect on future injury risk, as injury history is commonly cited as the greatest predictor of future injury risk [79]. Concerningly, the prevalence of concussion and non-concussion injury history in youth sports is high in some contexts. For example, 34% of girl and 23% of boy youth rugby players reported an injury within the past 12 months [80], 32% of Gaelic Games adolescent boys became injured in one season [81] and 35% of girl (U16–U18) Australian Rules football players reported a history of serious concussion (defined as knocked out or loss of consciousness) [82]. Given that such a high proportion of injuries in collision sports occur in contact events within the game, by offsetting the age of contact introduction, we could anticipate a reduction in injuries, reducing younger athletes’ risk for future injury.

### Reduction in Head Contact

In recent years, a greater emphasis has been placed on the quantification and prevention of head impacts and/or head acceleration events (HAEs) broadly, as opposed to just concussions alone. Although not all head contacts or HAEs lead to a concussion, efforts to reduce the accumulation of HAEs are seen as beneficial in two ways: firstly, to limit the opportunities for concussion to occur, and secondly, to limit the long-term exposure to HAEs and any potential associated negative consequences [83]. For example, policy changes disallowing body-checking in ice hockey have reduced the number of both head contacts and concussions [56], though the effect of reducing HAEs on long-term outcomes is not yet known.

To demonstrate the potential implications of delaying an athlete’s first exposure to contact in collision sport, we can estimate the annual reduction in HAEs for players if contact introduction rules were modified, using an example from rugby union [84]. Importantly, these are illustrative estimates only and it is recognised that not all head contacts would be eradicated by the removal of the tackle, as illustrated by tackles accounting for 60% of all HAEs, and the presence of fewer but not zero head impacts in flag versus tackle football [85].

Two illustrative examples of the reduction in HAEs for rugby union players if contact introduction rules were modified are provided below (data summed from Fig. 4 in Bussey et al. [84])

Here, one can see the potential benefit of delaying the introduction of contact, leading to a reduction in overall HAEs, and likely concussion risk (i.e., for a player who otherwise would start at 11 years old, 1-year delay = 236 fewer HAEs, 2-year delay = 472 fewer HAEs, 3-year delay = 739 fewer HAEs, 4-year delay = 1006 fewer HAEs. However, this a complex issue: should impact frequency decrease but impact magnitude increase, such a change may lead to unintended consequences across other outcome measures (e.g., diagnosed concussions). While the rates of head contact may vary between environments and sports, even reductions which might be considered small may be meaningful over the span of a career and could be considered a beneficial change, provided there is no associated increased risk of higher magnitude impacts.

### Previous Experience May Not Protect Against Injury

Previous experience with a collision sport is often anecdotally reported to reduce injury risk, therefore supporting an early introduction to collision sports. For example, limited contact experience amongst girls starting to play rugby is often cited as a reason for the higher rates of injury in girls compared to their boy counterparts [79, 80]. In some sports, many advocate for earlier introduction to contact to avoid such an increase in injury risk when contact is introduced at a later age. Yet, when examining the limited literature across sports, previous experience has been found to have no significant influence on injury outcomes in rugby union [80, 86, 87] or ice hockey [88], and inconsistent associations with injury outcomes in Gaelic Games [89, 90]. Shill et al. [80] found no differences in risk for injury, concussion, or tackle related concussion injuries in either boys or girls irrespective of level of experience. Further, Eliason et al. [88] concluded that disallowing body checking in games (while promoting progressive body-checking skill development in training) in youth ice hockey did not have unintended consequences related to injury and concussion rates when those athletes were later allowed to body check in games. Importantly, another area of inconsistency is the way in which player experience and contact experience are assessed as risk factors, with surrogate measures including age, age introduced to the sport [86], previous experience in years [80], or body-checking experience [88] reported. Similarly, very few studies have examined how differences in experience within the sport may influence girls and boys differently. Finally, the interplay between number of previous years’ experience and likelihood of injury (significantly associated with future injury [79]) must be recognised, with earlier exposure leading to a potentially earlier injury, and therefore potentially a greater injury risk in the future.

## Benefits of Introducing Contact at an Earlier Age

### Participation Rates

As with introducing contact at a later age, participation rates may grow with an earlier introduction to contact when combined with progressive modified rules and a skills training program. Player retention in collision sports may be enhanced if they have completed such a program from a young age and feel confident to enter full-contact games, especially in the context of adolescence when competitiveness increases and differences in size and athleticism emerge. Preserving the traditional aspects of collision sports throughout all levels of play appeals to many, and may benefit participation rates, cultural support and viewership [91, 92].

### Increased Skill Acquisition and Duty of Care

As stated previously, the ability to make contact is a movement skill, which can be acquired, developed, and mastered to protect the player and enhance participation in the sport [93]. From a skill-learning perspective, the point of entry into collision sports for children and adolescents should not be a question of age, but one of competence to safely perform the techniques and manoeuvres required of the sport. Sporting bodies, coaches and parents have a duty of care to provide a training environment that provides adequate skill development and prepares the players for the inevitable demands of collision sport. Sports that include contact skills requiring a high level of technical skill development could consider early progressive contact skills in training (with or without concurrent modified match contact, progressing to full) [94]. The decision to include concurrent match contact may be sport-specific and depend on the frequency and magnitude of head impact risk.

### An Early Progressive Approach

Exposure to a range of movement experiences early on may develop skill capacities that will facilitate the learning of more advanced skills. Significant developmental improvements in cognitive processes, such as processing speed (reaction time) and executive function, occur between the ages of 5 and 7 years, and children become more interested in structured rule-bound play [95–98]. Considering the improvements in cognitive processes and executive function at this developmental stage, the contact skills required for safe participation in collision can be introduced between the ages of 7 and 11 years. Before any sport-specific techniques are introduced, players would need to prepare and condition themselves for contact through performing fundamental movement skills such as falling, grappling and wrestling. These fundamental movements serve to prepare players for contact, for example, how to break a fall or physically engage (push, pull, drive, letting go) another player. For sport-specific development, players can move from a low-speed controlled structured environment to a more unstructured, dynamic and representative environment [93], as is the case in some of the existing body-checking skills-training programs in place in certain contexts [94].

From a coaching perspective, an understanding of game behaviors (demands) appropriate for that age group is required, along with knowledge of how to manipulate constraints to achieve specific contact skill-learning objectives [93, 99]. For example, in children’s team sports, the ‘beehive effect’ is typically observed where most players follow the ball, occupying only a small area of the playing field. The ‘beehive effect’ game dynamic suggests that contact intensity in collision sport may be less in the junior cohorts. For example, in rugby union the ‘beehive effect’ translates into more passive, jersey, arm and pulling tackles [100]. In terms of manipulating constraints, coaches can reduce the space of the playing field to reduce contact speeds and introduce simple rules to reduce direct contact with another player (e.g., players must evade opposing players, no direct running at the opposition). In the beginning, general game play (passing, running, positioning), forms of tag/touch rugby, can be separated from contact-focused games.

Of course, it is important to note that the rate of development greatly varies between children, and a range of physiological, psychological and social factors and experiences (beyond the scope of this article) impact skill learning and the capacity to learn skill during early childhood. While applying skill acquisition principles to the development of contact skills is slowly emerging [93], research on the application of learning theories and how it relates to the introduction of contact skills and the development thereof is almost non-existent. This not only highlights an avenue for future research, but an important consideration in the discussion of when to introduce contact in collision sports.

## Conclusion

The age at which contact (e.g., collision, tackling, body checking, shouldering, cross-checking) should be introduced to youth collision sport is a complex issue and often results in heated debate. While this can be polarising, we must consider the context of restrictions in sport more broadly, whereby modifying sport is not uncommon. Bowling limits (e.g., cricket), field size restrictions (e.g., American Football, rugby), restrictions on performance of certain movements (e.g., figure skating, gymnastics), and weight classification (e.g., mixed martial arts, boxing, wrestling) are examples of guidelines which, after some initial debate, have been readily accepted as means to protect athlete health. There will always be sport-specific advocates for and against any such policies, but athlete welfare and the protection of child and adolescent well-being are always the priority, irrespective of on which side of the debate one lies. When considering ‘At what age should contact be introduced?’, this article has summarised the key factors. To illustrate how complex this question is, other recent papers have suggested a blanket rule across sports to introduce tackling at 12 years old [101], yet the authorship group of the current paper could not agree on a recommendation for early or late introduction to contact. This highlights the need for future high-quality research and evidence-informed policy. What was generally acknowledged and agreed on was that introduction to contact should be a progressive and clearly defined process, with physiological, psychological and biomechanical competence and self-efficacy required to perform contact safely and optimally. Training of contact skills is a critical aspect of player welfare and delaying in-competition contact until this competence has been achieved is suggested. Although this issue is nuanced and the use of one-size-fits-all approaches to mandates often does not achieve the intended outcomes [102], the need for agreement and consensus on optimal strategies and their implementation for introducing deliberate contact and collision to youth sport would be a major step forward for collision sport.

## Supplementary Information

Below is the link to the electronic supplementary material.

Supplementary file1 (DOCX 29 KB)
