# Supplementary material for: The Contact Conundrum: Are We Introducing Contact at the Correct Time in Youth Sports?
Source: Sports Med. 2025 Dec 4;56(3):607–18. doi: 10.1007/s40279-025-02348-6 (PMC13017993; doi:10.1007/s40279-025-02348-6)
Supplement: Supplementary file 1 — Supplementary file1 (DOCX 29 KB) [file 40279_2025_2348_MOESM1_ESM.docx]

| **Table S1: Current Guidelines for Contact Introduction** | | | | | | | | | |
| --- | --- | --- | --- | --- | --- | --- | --- | --- | --- |
|  | **Country** | **Sex** | **Age** | **Level of contact** | **Conditions** | **Sanction** | **Mixed age categories** | **Age grade combinations permitted** | **Link** |
| Rugby Union | Ireland | Male/female | U8 | Tackle introduced | Waist high | Penalty. | From U12 and above mixed rugby no longer permitted | In the event of insufficient playing numbers, players can play across two age categories. | <https://d19fc3vd0ojo3m.cloudfront.net/irfu/wp-content/uploads/2022/09/02095557/IRFU-Mini-and-Rugby-Prime-Leprechaun-Rugby-Regulations-2022-2023.pdf> |
| Rugby Union | England | Male/female | U9 | Tackle introduced | Tackle including hold (i.e., when BC is sufficiently slowed but tackler unable to bring BC to ground). | NR | From U12 and above mixed rugby no longer permitted | **Males:** Certain age grades are permitted to play down one or two age grades for training and playing if recommended by the player’s club, as set out in the table at Regulation 15.5.  **Females:** Dual age banding is permitted in girls’ rugby due the minimal difference in the physical capabilities of female players in these bands. | [https://www.englandrugby.com//dxdam/fa/fa1dbfe2-9a2b-4171-9ee8-259313647fc5/AG%20Flyer%202019.pdf](https://www.englandrugby.com/dxdam/fa/fa1dbfe2-9a2b-4171-9ee8-259313647fc5/AG%20Flyer%202019.pdf) |
| Rugby Union | Wales | Male/female | U9 | Tackle introduced | Waist high tackle. 1v1 tackles only. Opposition receive the ball after the 6th tackle. | NR | From U12 and above mixed rugby no longer permitted | Players can only compete one year up. | <https://www.wrugamelocker.wales/en/pathway-laws/u9/> |
| Rugby Union | Scotland | Male/female | U9 | Tackle introduced | Waist high tackle. Tackle hold (i.e., when BC is sufficiently slowed but tackler unable to bring BC to ground). 1v1 tackles only. No targeting of the ball | Free pass to non-offending team. | From U12 and above mixed rugby no longer permitted | Youth rugby can be played in a maximum of 2-Year age bandings. | <https://scottishrugby.org/wp-content/uploads/2023/08/RD_BLV_P4.pdf> |
| Rugby Union | Australia | Male/female | U8 | Tackle introduced | Jersey and sling tackles prohibited. Fending to the head and squeeze ball illegal. | NR | NR | NR | <https://d26phqdbpt0w91.cloudfront.net/NonVideo/e5b040a3-7fea-4f4c-c0f5-08d976418c32.pdf> |
| Rugby Union | New Zealand | Male/female | U8 | Tackle introduced | Two sessions must be completed before the season starts. A ‘tackle clinic’ must be completed by all the U8 coaches before the season starts. Tackle below the sternum. | Penalty. | Single gender teams after U13. | Players can play one year up. In exceptional circumstances, case approval is required for those seeking to play 2 years up. | <https://www.nzrugby.co.nz/assets/DSLV-2024-LR_compressed-002.pdf> |
| Rugby Union | Canada | Male/female | U11 | Tackle introduced | Below waist tackles, ball cannot be ripped. | Penalty. | A player over 12 but under 15 years of age may play on a mixed gender contact rugby team where no other option exists. | NR | <https://rugby.ca/uploads/Community/Age_Grade_Law_Variations_2023/English/Rugby_Canada_-_2022_Law_Variations_Community_Rugby_Eng_U11_U12_v3_Jan2023.pdf> |
| Rugby Union | South Africa | Male/female | U10 | Tackle introduced | Below the base of the sternum and above the knees. Sling tackles are prohibited. | Penalty. | NR | In primary School Rugby, no player more than two years younger than the prescribed maximum stipulated age within an age-grade  category, may participate within that age‐grade during the year in question. The player may not be older than the stipulated age‐grade. | <https://www.sareferees.co.za/media/f1wi4xrw/primary-school-final-law-variations-2024.pdf> |
| Rugby Union | Japan | Male/female | U12 | Tackle introduced | Tackle below the sternum. | Penalty. | Mixed play up until U15 age groups. | NR | https://rugby-japan.s3.ap-northeast-1.amazonaws.com/file/html/164383_64559cb8ad8a4.pdf |
| Rugby League | England | Male/female | U6 | Tackle introduced | Tackle is allowed if agreed by coaches. Tackles below the armpit. 6 tackle games. | Penalty. | Up to U11 age group the teams are mixed with boys and girls playing together. | NR | <https://www.rugby-league.com/ages-and-stages/core/u6-u9> |
| Rugby League | New Zealand | Male/female | U6 | Tackle introduced | Tackle below the armpit. 4 tackle games. No stripping or stealing of the ball. | Penalty. | The maximum age for males and females to play in mixed gender full contact Rugby League is 12 years of age. | NR | <https://nzrl.co.nz/wp-content/uploads/2020/05/NZRL-Mini-Mod-Rules.pdf> |
| Rugby League | Australia | Male/female | U8 | Tackle introduced | Tackle below the armpit. 6 tackle games. No stripping or stealing of the ball. | Penalty. | Males and females can participate in Mixed Gender Rugby League Competitions up to the age of 12 years. | 18 month registration policy. | <https://www.playrugbyleague.com/laws-of-the-game-community/junior-league-laws-6-12s/> |
| Australian Football | Australia (national guidelines) | Male/Female | U9 | Modified tackling introducing (wrap) | No pushing, bumping or barging. | Free kick to non-offending team. | For the avoidance of doubt, mixed Competition age groups are only permitted up to and including Under 14. | A Player may apply for dispensation to play in a Competition age group below their applicable age group on the basis of a Disability or for Physical Size Considerations by submitting an application in accordance with Section 4.1(b). | <https://play.afl/sites/default/files/2024-06/JuniorRules_May24_Final.pdf> |
| Australian Football | Australia (national guidelines) | Male/Female | U11 | Full tackling, bumping and barging introduced | Full tackling, bumping and barging permitted | Free kick to non-offending team. | For the avoidance of doubt, mixed Competition age groups are only permitted up to and including Under 14. | A Player may apply for dispensation to play in a Competition age group below their applicable age group on the basis of a Disability or for Physical Size Considerations by submitting an application in accordance with Section 4.1(b). | <https://play.afl/sites/default/files/2024-06/JuniorRules_May24_Final.pdf> |
| Australian Football | Australia (Yarra Junior Football) | Male/Female | U10 | Full tackling, bumping and barging introduced | Full tackling, bumping and barging permitted in the mixed U10 competitions | Free kick to non-offending team. | For the avoidance of doubt, mixed Competition age groups are only permitted up to and including Under 14. | NR | <https://www.yarrajfl.org.au/rules-by-laws> |
| Australian Football | Australia (Yarra Junior Football) | Male/Female | U10 | Modified tackling introduced | Modified Tackling permitted – A player in possession of the ball may be tackled by an opponent  wrapping both arms around the area below the top of the shoulders and on/above the  knees. The tackle may be from either side or from behind, providing the tackle from  behind does not thrust the player with the ball forward (i.e., push the player in the back). No bumping/barging permitted. | Free kick to non-offending team. | For the avoidance of doubt, mixed Competition age groups are only permitted up to and including Under 14. | NR | <https://www.yarrajfl.org.au/rules-by-laws> |
| Australian Football | Australia (Yarra Junior Football) | Female | U11 | Full tackling introduced | Full tackling, bumping and barging permitted. | Free kick to non-offending team. | For the avoidance of doubt, mixed Competition age groups are only permitted up to and including Under 14. | NR | <https://www.yarrajfl.org.au/rules-by-laws> |
| Gaelic Football | Ireland | Male | No age restriction | Tackle introduced  Shoulder charge introduced | Near hand tackle, aimed at the ball, not the player.  Provided a player has at least one foot on the ground, a player may make a shoulder to shoulder charge on an opponent (a) who is in possession of the ball, or (b) who is playing the ball other than when kicking it, or (c) when both players are moving in the direction of the ball to play it. | Free to non-offending team. | Girls may participate only up to and including the U12 grade. | Players can play up to two age grades above at the U12 and lower age grades e.g. an under 8 can play their own age grade and under 9 or under 10 level. | <https://www.gaa.ie/api/pdfs/image/upload/vxpgnvzwyiogaylcqubh.pdf> |
| Ladies Gaelic Football | Ireland | Female | NA | None | No deliberate body contact. | Free to non-offending team. | Boys may participate only up to and including the under 12 grade. | Players can play up to two age grades above at the under 12 and lower age grades e.g. an under 8 can play their own age grade and under 9 or under 10 level. | https://ladiesgaelic.ie/wp-content/uploads/2018/03/LGFA-Playing-Rules-2024.pdf |
| Hurling | Ireland | Male | No age restriction | Shoulder charge introduced | Provided a player has at least one foot on the ground, a player may make a shoulder to shoulder charge on an opponent (a) who is in possession of the ball, or (b) who is playing the ball, or (c) when both players are moving in the direction of the ball to play it. | Free to non-offending team. | Girls may participate only up to and including the U12 grade. | Players can play up to two age grades above at the under 12 and lower age grades e.g. an under 8 can play their own age grade and under 9 or under 10 level. | <https://www.gaa.ie/api/pdfs/image/upload/vxpgnvzwyiogaylcqubh.pdf> |
| Camogie | Ireland | Female | No age restriction | Shoulder charge introduced | A player must not: Deliberately shoulder an opponent other than shoulder to shoulder. | Free to non-offending team. | Boys may participate only up to an including the under 12 grade. | Players can play up to two age grades above at the under 12 and lower age grades e.g. an under 8 can play their own age grade and under 9 or under 10 level. | https://camogie.ie/wp-content/uploads/2024/09/Part-2-Official-Rules-30-09-24-A4-web.pdf |
| Field Lacrosse | North America | Male | U15 | Cross checking, body checking introduced | Body checking is prohibited when the opponent is not in possession of the ball or within three yards of it, when the check is from behind, below the waist, or above the shoulders, when the opponent is off the ground, or when it targets vulnerable players, including those with their head down, turning away to receive a pass, | Penalty | NR | NR | <https://www.usalacrosse.com/sites/default/files/documents/Rules/2022-boys-youth-rulebook.pdf> |
| Field Lacrosse | North America | Female | U12 | Modified checking introduced | Modified checking is checking where the checking movement solely occurs below the shoulder of the player with the ball. The check must be down and away from the body. | No stick to stick, body to body, or body to stick contact- results in a foul or removal from play. | NR | NR | https://www.usalacrosse.com/sites/default/files/documents/Rules/2024-girls-youth-guidebook.pdf |
| Box Lacrosse | Canada | Male | No age restriction | Body checking | No restrictions stated in rulebook for male leagues. Body checking from behind is prohibited at all levels of play. | Penalty | NR | NR | https://cloud.rampinteractive.com/canadianlacrosse/files/misc%20pdf/2019_-_2020_box__rule_%26_situation_handbook.pdf |
|  |  | Male | No age restriction | Cross Checking  (two-handed stick push) | Cross checking is permitted on the ball carrier to push an opponent away.  Cross checking is permitted within the “house” dotted line on the non-ball carriers in Pee Wee [U13)] and lower levels. | Penalty | NR | NR | <https://www.bclacrosse.com/pdfs/2017%20CLA%20Rulebook%20APPENDIX%20H%20-%20Female%20Novice%20Rules.pdf>  (pages 140 – 153) |
|  |  | Female | U15 | Body checking | Body checking is permitted.  Body checking from behind is prohibited at all levels of play. | Penalty | NR | NR | <https://www.bclacrosse.com/pdfs/2017%20CLA%20Rulebook%20APPENDIX%20H%20-%20Female%20Novice%20Rules.pdf>  (pages 156 – 159) |
|  |  | Female | U11 | Cross Checking  (two-handed stick push) | Cross checking contact is permitted on the ball carrier at any time with use of equal pressure to prevent her movement. Cross checking is permitted only on non-ball carriers within the “house” dotted line and the contact may only be equal pressure against the opponent to prevent her movement. | Penalty | NR | NR | <https://www.bclacrosse.com/pdfs/2017%20CLA%20Rulebook%20APPENDIX%20H%20-%20Female%20Novice%20Rules.pdf>  (pages 156 – 159) |
| Ice Hockey | Canada | Female | NA | None | Intentional body checks prohibited | Penalty | NR | NR | http://rulebook.hockeycanada.ca/english/part-ii-gameplay-fouls/section-7/rule-7-3/#:~:text=A%20Minor%20penalty%20will%20be,a%20Member%20of%20Hockey%20Canada. |
| Ice Hockey | Canada | Male | U15 | Body checking introduced | An attempt by a player to gain an advantage on their opponent with the deliberate use of the body. | Penalty | NR | NR | http://rulebook.hockeycanada.ca/english/part-ii-gameplay-fouls/section-7/rule-7-3/#:~:text=A%20Minor%20penalty%20will%20be,a%20Member%20of%20Hockey%20Canada. |
| Ice Hockey | USA | Females | NA | None | Intentional body checks prohibited | Penalty | NR | NR | https://www.usahockeyrulebook.com/page/show/1084648-rule-604-body-checking |
| Ice Hockey | USA | Males | U15 | Body checking introduced | In Body Checking categories, the primary focus of the check shall be to gain possession of the puck and officials should strictly penalise any illegal actions such as boarding, charging, cross checking, and a late body check to a player who is no longer in control of the puck. | penalty | NR | U14 players cannot participate in higher age divisions | <https://cdn1.sportngin.com/attachments/document/f3f4-2473110/Junior_Rulebook_2125_FINAL.pdf?_gl=1*1npsu7y*_ga*MTU2MTY1Mzc2MC4xNzMzNDAwMzky*_ga_PQ25JN9PJ8*MTczMzQwMDM5Mi4xLjEuMTczMzQwMDQxMy4wLjAuMA..#_ga=2.268443377.1012602803.1733400399-1561653760.1733400392> |
| Ice Hockey | England | Male/Female | U15 | Body checking introducing. | A player can use a shoulder, hip or torso to hit or impede an opponent, but only when the opponent is in possession of the puck. | Penalty | All junior age group leagues (U9-U18) are mixed gender. | There is free movement of players to play up within their primary club. Due diligence is necessary to ensure the player is safe and capable both physically  and mentally of playing up an age group. A female player who is aged 10 (or 9 if a netminder) on the date of the fixture may  play up to any division in the girls’ U16 division. | https://englandicehockey.com/wp-content/uploads/2024/08/Junior-Rules-of-Competition-2024-2025.pdf |
| American Football | America (Pop warner tackle football) | Male/Female | U7 | 11-man football: tackle introduced at 'tiny mite' level- ages 5-6-7 | In addition to other specific prohibitions in the National  Federation and NCAA rule books, no butt blocking, chop blocking,  clipping, face tackling or spearing techniques are permitted in Pop  Warner. Contact training: No full-speed head-on blocking or tackling drills in which the  players line up more than 3 yards apart are permitted. Max 30 mins contact per day. | Penalty | All Pop Warner programs and activities are open to youth – males and females | Operation of age/weight schematics- the traditional age/weight schematic provides a uniform series of three basic years of age qualification for each level of play, with a fourth year of eligibility falling under more stringent weight restrictions (the “older/lighter” player). The “older/lighter” player is indicated by an asterisk. (See Rule 1, S4) | https://dt5602vnjxv0c.cloudfront.net/portals/21799/docs/rule%20books/24-pw-002_popwarner-rulebook-football_4.25x9.25__digital_final_compressed_v2.pdf |
| Canadian Football | Canada | Male | U11 | Tackle introduced | NR | NR | NR | NR | <https://footballcanada.com/news/2019-football-canada-u8-mandate/> |
| NR not reported, NA not applicable | | | | | | | | | |
